# Phenotypic Diversity of Multicellular Filamentation in Oral Streptococci

**DOI:** 10.1371/journal.pone.0076221

**Published:** 2013-09-27

**Authors:** Valentina Rossetti, Thomas W. Ammann, Thomas Thurnheer, Homayoun C. Bagheri, Georgios N. Belibasakis

**Affiliations:** 1 Institute of Evolutionary Biology and Environmental Studies, University of Zurich, Zurich, Switzerland; 2 Institute of Oral Biology, Section for Oral Microbiology and Immunology, University of Zurich, Zurich, Switzerland; 3 Anthropological Institute and Museum, University of Zurich, Zurich, Switzerland; University of Toronto, Canada

## Abstract

Filamentous multicellular bacteria are among the most ancient multicellular organisms. They inhabit a great variety of environments and are present in the human body, including the oral cavity. Beside the selective advantages related to the larger size achieved through filamentation, the development of multicellular bacteria can be also driven by simple ecological factors such as birth and death rates at the cellular level. In order to extend earlier results obtained in aquatic species, we investigate the filamentation process of four different strains of oral streptococci, namely *S. mutans*, *S.* salivarius, *S.* oralis and *S. anginosus*. The results indicate differences in the capacities of different streptococcus species to form filaments, manifested in terms of length and the time-scale of filament elongation. The filamentation pattern of these oral streptococci resembles that of aquatic bacteria, whereby filaments reach a peak length during exponential growth and become short when the population reaches a steady state. Hence, this study validates that multicellularity can be an emergent property of filamentous bacteria of different ecological niches, and that phenotypic differences in filamentation can occur within species of the same genus, in this case oral streptococci. Moreover, given the role that specific oral streptococci can play in the etiology of oral diseases, these results can possibly open new perspectives in the study of the virulence properties of these species.

## Introduction

Filamentous multicellular bacteria are highly prevalent in diverse ecosystems spanning from water basins, soil and extreme environments [1-3] to the human body [4,5] and oral cavity [6-8]. Typically formed by several individual cells arranged in a chain [3,9,10], multicellular filamentous bacteria were among the first multicellular organisms inhabiting the planet [11]. The filamentous shape can imply size-related advantages, such as enhanced feeding abilities, better dispersal, and a lower risk of predation [12,13]. The same advantages can be also valid for single-celled bacteria that under specific conditions develop into oblong, filamentous single cells [14]. Moreover, filament-forming species can survive in hostile environments and are often associated with other bacteria in forming biofilms [2]. Multicellular filaments can also exhibit cellular differentiation, as is the case in some species of nitrogen-fixing cyanobacteria [15,16]. In this case, some cells of the filament are devoted to nitrogen fixation, while the other ones perform oxygenic photosynthesis. Theoretical studies showed also that in the specific case of cyanobacteria, multicellularity was a necessary condition for the evolution and optimization of division of labor within a single organism [17].

Beside the selective advantages related to morphology, it has been shown that multicellularity in aquatic bacteria can be an emergent property driven by ecological factors such as birth and death rates of cells [18]. In three heterotrophic species as well as in two cyanobacterial strains, it was found that the average filament length at different growth stages depends on the generation time of the species. This is in turn determined by their life-history traits. During the exponential phase, species with low cellular birth and death rates (corresponding to a long generation time) develop into much longer filaments than species with high rates, and hence shorter generation times. Nonetheless, all tested species showed a common temporal pattern, whereby after an initial phase characterized by short individuals, filaments reached their maximum elongation during the exponential phase and became short again once the population reached the carrying capacity. This is the phase in which the population does not grow anymore, and the birth rate equals the death rate. These experimental results were supported by a theoretical model, according to which strains with different generation time can still have the same fitness in terms of population growth.

The findings described above [18] denote that the evolution of multicellularity is an emergent property in aquatic species, and warrants further investigations on species living in different environmental niches. In this sense, the oral cavity can serve as a relevant example of a heavily colonized niche of the human body. Several oral bacteria tend to form long filaments which may also constitute part biofilms. These are complex polymicrobial biofilm communities attached on the surfaces of the teeth or the soft oral mucosa, with physiological relevance to oral health or disease [19]. Members of the genus *Streptococcus* are the major inhabitants of the oral cavity [20,21]. The four major groups of oral streptococci include the species groups *salivarius*, *anginosus*, *mutans* and *mitis* [22]. One strain of each group was selected for this study, *S. salivarius* of the *salivarius* group, *S. anginosus* of the *anginosus* group, *S. mutans* of the *mutans* group, and *S. oralis* of the *mitis* group. While *S. mutans* is highly associated with the development of dental caries (tooth decay), species of the other groups including *S. anginosus*, *S.* salivarius and *S. oralis* have less distinctive roles in oral disease, and may be more associated with oral health. Differences in the pathogenicity of the various streptococcus groups could be attributed to their metabolism, and potentially, their capacity to multiply and form filamentous structures. Little is known about the capacities of different oral streptococcus species to form filaments. The aim of this study was to investigate in an *in vitro* experimental model the capacity of four oral streptococcus species in their capacity to form filaments over time. The hypothesis is that similarly to the observations made earlier in aquatic organisms, the four examined oral streptococci differ in their capacity to form filaments, which could potentially impact their virulence properties.

## Material and Methods

### Cultures of Streptococci

The species used in this study were *S. oralis* SK248 (OMZ 607), *S. salivarius* (OMZ 036), *S. mutans* ATCC 700610 (OMZ 918) and *S. anginosus* ATCC 9895 (OMZ 871). All strains were maintained on Columbia Blood Agar (CBA) plates. Prior to the experiments, each strain was transferred into 10 ml of modified fluid universal medium (mFUM) [23] and incubated overnight to reach the stationary phase. The protocol was identical for all four strains.

Prior to the start of the growth experiments, 2.5 ml of the precultures were diluted in 72.5 ml of mFUM and vortexed. The diluted starting culture was distributed into 15 individual tubes each containing 5 ml, and incubated anaerobically at 37°C. Two individual experiments have been performed. Analytical results in the following sections are shown for one of the two experiments.

### Preparation of cultures for microscopy and imaging

Each of the 15 prepared tubes was sampled to generate the data for one time point of the growth curves. The tubes were put on ice immediately after removing them from the incubator and after finishing the below described steps stored at -20°C. 1 ml of the culture was used for the determination of optical density at 550 nm (OD_550_). If the OD_550_ was above 0.9, the culture was diluted in order to reach a density below this value. 2 ml of the original culture were transferred into an Eppendorf tube and centrifuged (5 min, 20’000x g, 4°C). The supernatant was removed and replaced with 2 ml of 4% *para*-formaldehyde (PFA). After at least 1 h of fixation, the samples were mounted on 24-well slides for microscopy. To this end, the samples were serially diluted in coating buffer consisting of 0.00025% Cetyltrimethylammonium bromide (CTAB, Sigma-Aldrich, Buchs, Switzerland) in 0.9% NaCl with the addition of 0.02% NaN_3_ to maintain sterility during storage. 10 µl of the samples were then pipetted onto the wells and the drops were air dried at room temperature. After drying, the slides were fixed for 2 min in MeOH. Thereafter, the samples were stained during 20 min at 100% humidity using 8 µl per well of a mixture of 3 µM Yo-Pro-1 iodide (Invitrogen, Zug, Switzerland) and 15 µM Sytox Green (Invitrogen). After removing the staining solution and washing the slides in distilled water, 100 µl of mounting fluid (90% glycerol ultrapure (Invitrogen), 10% 25 mg g^-1^ 1,4-diazabicyclo[2.2.2]octane (DABCO, 98%, Sigma-Aldrich) in 10x PBS) were pipetted onto the slides prior to covering them with cover glasses. For the evaluation of each time point, two individual wells were prepared, and three images were taken at random spots in each well using a Olympus E-510 camera (Olympus Optical, Volketswil, Switzerland) mounted on a Olympus BX60 microscope (Olympus Optical) with a x100 oil immersion objective (numerical aperture: 1.35). For each time point, we hence measured filament length by analyzing six distinct pictures coming from two different wells.

### Analysis of microscopy images

The length of each filament was determined by manual measurement using the Soft Imaging software CellF (Olympus, Germany). For each time point and species, the length of at least 70 filaments was measured (Table 1). Plots were elaborated with Matlab (The MathWorks Inc., Natick, Massachusetts). The cell length was calculated individually for each species by averaging the length of at least 60 cells, at different time points.

**Table 1:** Maximum and average length measured in number of cells.

***S. mutans***						***S. salivarius***				
Time (h)	Nr. Of measures		Min-Max length (cells)		Average length (cells)	Time (h)	Nr. Of measures	Min-Max length (cells)		Average length (cells)
0.0	106		0.6	11.0	3.2	0.0	78	0.6	9.4	3.1
1.0	95		0.8	10.8	4.1	1.0	103	0.6	19.3	4.2
1.6	80		1.0	14.5	4.3	2.9	217	0.1	28.6	6.0
**2.2**	125		0.7	**15.1**	**4.4**	**4.0**	145	1.1	30.2	**8.9**
3.0	93		0.7	12.6	4.1	4.5	135	1.2	46.8	7.0
4.0	108		0.5	13.3	3.0	4.8	138	1.0	36.7	8.3
5.0	144		1.0	12.2	3.1	5.2	192	1.0	28.7	6.0
6.0	77		0.7	9.2	3.0	5.9	112	0.9	45.4	8.3
6.3	149		0.8	10.7	2.1	6.7	147	1.1	40.6	7.8
6.8	155		0.6	7.7	2.3	7.4	126	1.1	23.7	6.7
7.3	125		0.7	7.8	1.9	**8.2**	197	0.8	**50.9**	8.4
7.8	132		0.7	7.5	2.3	9.2	112	1.1	25.2	6.1
8.3	206		0.4	8.6	1.9	26.2	184	0.8	19.4	4.4
23.6	210		0.5	7.3	1.8					
27.6	136		0.6	7.0	1.8					
***S. anginosus***						***S. oralis***				
Time (h)		Nr. of measures	Min-Max length (cells)		Average length (cells)	Time (h)	Nr. of measures	Min-Max length (cells)		Average length (cells)
0.0		101	1.4	16.8	4.0	0.0	102	1.0	16.3	3.7
0.9		209	0.9	11.7	3.9	0.9	123	0.8	12.3	3.6
**1.3**		150	1.1	14.3	**4.5**	**1.3**	153	0.7	9.9	**3.9**
2.1		193	0.7	14.5	3.8	2.1	216	0.6	11.9	3.6
2.7		193	0.5	14.9	4.3	2.7	147	0.4	9.7	2.2
3.1		187	0.7	13.5	3.5	3.1	163	0.2	9.6	2.1
**4.2**		125	0.7	**16.3**	3.5	3.7	107	0.8	10.7	2.8
5.2		177	0.8	13.1	3.5	4.2	198	0.7	17.4	2.5
**7.8**		182	0.9	14.3	**4.6**	5.5	132	1.0	10.1	2.5
10.0		102	0.7	10.5	3.7	7.8	150	0.8	6.3	2.4
12.2		205	0.4	10.4	2.8	8.9	189	1.0	6.9	2.5
73.0		172	0.4	8.3	1.8	10.0	89	0.7	5.4	2.5
						12.2	287	1.1	17.2	3.2
						**73.0**	224	0.5	**18.2**	3.1

Results are derived from one representative experiment.

### Statistical Methods

We checked the difference in filamentation across species by mean of a statistical comparison of the length dataset in terms of number of cells at five reference measurement time points. For the statistical analysis only one of the two experiments was chosen as representative, and therefore the presented data is derived from intra-experimental replicates. Since the number of measurement time points is not the same for all strains, we picked those corresponding to the first point after inoculum, the early exponential phase, the middle time point, the late growth phase and the end point of the experiment. Expressed in hours after inoculum, these are 1, 2, 5, 7.3 and 27.6 hours for *S. mutans*, 1, 4, 4.8, 7.4 and 26 hours for *S. salivarius*, 1, 2, 3, 7.75 and 73 hours for both *S. anginosus* and *S. oralis*.

The statistical procedure that we used compares the four strains initially with a Kruskal-Wallis test at the 0.05 significance level. The choice of a non-parametric method is supported by the fact that the data are not normally distributed. However, the Kruskal-Wallis test for the comparison of multiple samples only indicates whether there is at least one sample that differs from the others in terms of median. In order to get statistical support for the differences between single pairs of strains, we used the results of the Kruskal-Wallis test to perform a multiple comparison that implements the Tukey’s honestly significant difference criterion. The output of the multiple comparison is expressed in Table 2. This indicates the pair compared (columns 1 and 2), the estimated difference in rank (column 4) and the 95% confidence interval of the difference in rank (columns 5 and 6). If the extremes of the confidence interval have the same sign (i.e. the interval does not contain the value 0), the samples are significantly different at the 0.05 level. To facilitate the reading of the table, the third column indicates if the considered pair is significantly different or not. The result of the Kruskal-Wallis test is shown in column 7. Both the Kruskal-Wallis and the multiple comparison procedures were performed with the statistical package of Matlab (The MathWorks Inc., Natick, Massachusetts).

**Table 2:** Results of the statistical procedures for the comparison of strains at different time points, derived from one experiment: multiple comparison with Tukey’s honestly significant difference criterion (columns 1-6) and Kruskal-Wallis method (column 7).

**Early time point: S. mutans: 1h, S.salivarius: 1h, S. anginosus: 1h, S. oralis 1h**						
Pair of species tested		Significant difference	Estimated difference in rank	95% confidence interval of the difference in rank		Kruskal-Wallis
*S. mutans*	*S. salivarius*	no	0.65	-56.22	57.52	p=0.3>0.05
*S. mutans*	*S. anginosus*	no	1.20	-53.91	56.30	
*S. mutans*	*S. oralis*	no	35.70	-25.13	96.53	
*S. salivarius*	*S. anginosus*	no	0.55	-45.23	46.32	
*S. salivarius*	*S. oralis*	no	35.05	-17.47	87.58	
*S. anginosus*	*S. oralis*	no	34.50	-16.11	85.11	
**Early exponential phase: S. mutans: 2h, S.salivarius: 4h, S. anginosus: 2h, S. oralis: 2h**						
Pair of species tested		Significant difference	Estimated difference in rank	95% confidence interval of the difference in rank		Kruskal-Wallis
*S. mutans*	*S. salivarius*	**yes**	-186.30	-247.81	-124.80	p=0<0.05
*S. mutans*	*S. anginosus*	no	36.54	-21.31	94.40	
*S. mutans*	*S. oralis*	no	56.58	-0.06	113.21	
*S. salivarius*	*S. anginosus*	**yes**	222.85	167.47	278.23	
*S. salivarius*	*S. oralis*	**yes**	242.88	188.78	296.98	
*S. anginosus*	*S. oralis*	no	20.03	-29.88	69.95	
**Middle time: S. mutans: 5h, S.salivarius: 4.8h, S. anginosus: 3h, S. oralis: 3h**						
Pair of species tested		Significant difference	Estimated difference in rank	95% confidence interval of the difference in rank		Kruskal-Wallis
*S. mutans*	*S. salivarius*	**yes**	-201.33	-257.21	-145.45	p=0<0.05
*S. mutans*	*S. anginosus*	no	-30.03	-82.04	21.97	
*S. mutans*	*S. oralis*	**yes**	112.87	59.23	166.52	
*S. salivarius*	*S. anginosus*	**yes**	171.30	118.66	223.94	
*S. salivarius*	*S. oralis*	**yes**	314.20	259.94	368.47	
*S. anginosus*	*S. oralis*	**yes**	142.91	92.64	193.17	
**Late growth phase: S. mutans: 7.3h, S.salivarius: 7.4h, S. anginosus: 7.75h, S. oralis: 7.75h**						
Pair of species tested		Significant difference	Estimated difference in rank	95% confidence interval of the difference in rank		Kruskal-Wallis
*S. mutans*	*S. salivarius*	**yes**	-234.22	-288.80	-179.65	p=0<0.05
*S. mutans*	*S. anginosus*	**yes**	-188.82	-238.55	-139.09	
*S. mutans*	*S. oralis*	**yes**	-42.28	-94.39	9.84	
*S. salivarius*	*S. anginosus*	**no**	45.41	-7.32	98.13	
*S. salivarius*	*S. oralis*	**yes**	191.95	136.97	246.93	
*S. anginosus*	*S. oralis*	**yes**	146.54	96.37	196.72	
**End point: S. mutans: 27.6h, S.salivarius: 26h, S. anginosus: 73h, S. oralis: 73h**						
Pair of species tested		Significant difference	Estimated difference in rank	95% confidence interval of the difference in rank		Kruskal-Wallis
*S. mutans*	*S. salivarius*	**yes**	-283.69	-342.89	-224.49	p=0<0.05
*S. mutans*	*S. anginosus*	no	-22.45	-82.73	37.84	
*S. mutans*	*S. oralis*	**yes**	-193.30	-249.61	-136.99	
*S. salivarius*	*S. anginosus*	**yes**	261.24	199.07	323.42	
*S. salivarius*	*S. oralis*	**yes**	90.39	32.06	148.72	
*S. anginosus*	*S. oralis*	**yes**	-170.86	-230.29	-111.42	

## Results

**Figure 1:**
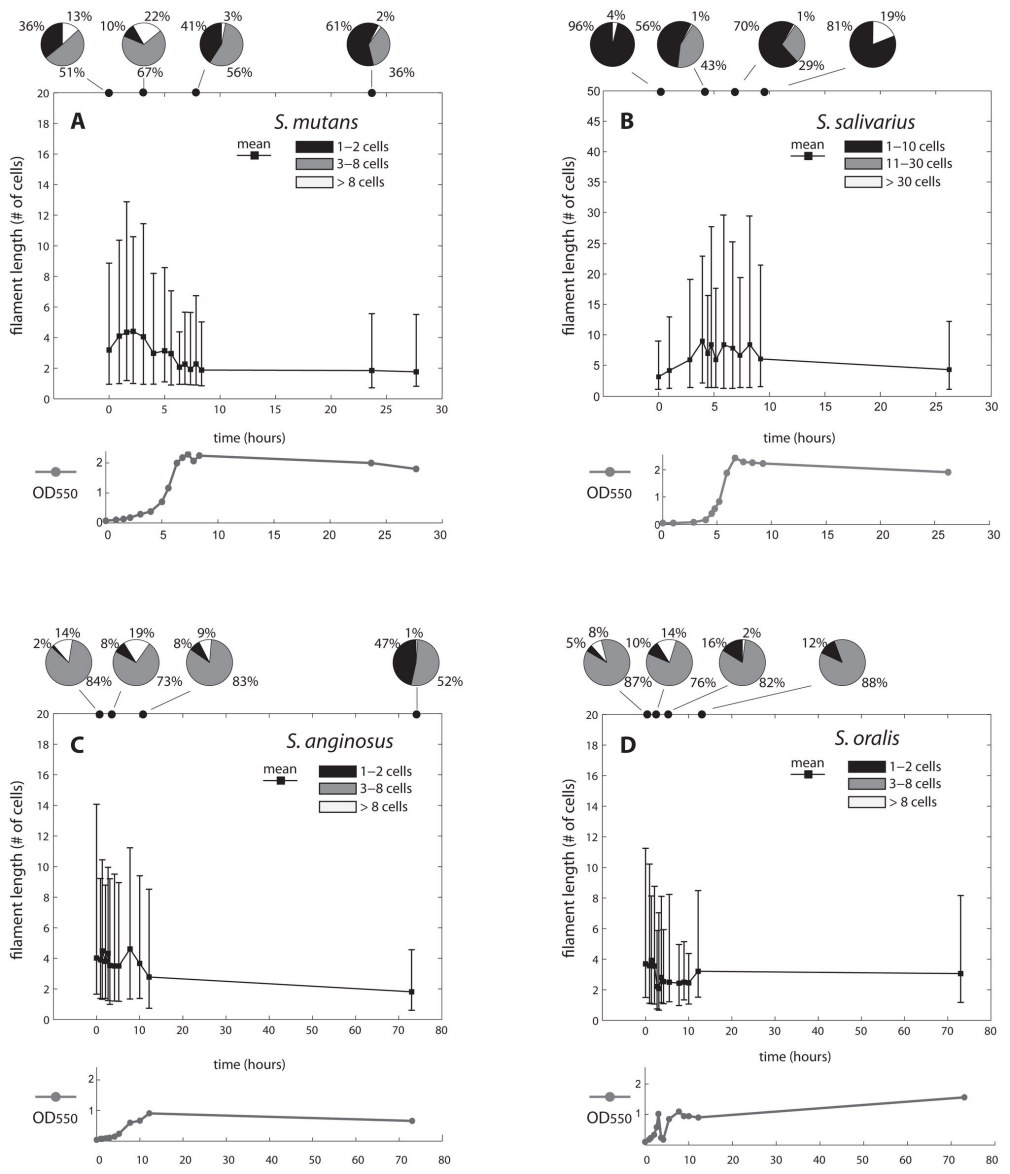
Mean filament length and growth curves at successive time points. The plots show the mean filament length (black line) and optical density (OD550) measurements (grey line) of *S. mutans*, *S. salivarius*, *S. anginosus* and *S. oralis* at successive time points during growth. Data are based on the analysis of six pictures coming from two distinct wells per time point, from one representative experiment. Length was calculated in terms of number of cells of the filaments. Lower and upper edges of the error bars indicate the 2.5th and 97.5th percentiles respectively. Pie charts on top of each plot indicate the distribution of short, medium and long filaments at four distinct growth phases. These categories correspond to 1-2, 3-8 and more than 8 cells in the case of *S*. *mutans*, *S*. anginosus and *S*. *oralis*. In the case of S. salivarius, they correspond to 1-10, 11-30 and more than 30 cells. The y-axis scale of *S*. *salivarius* differs from the others for a better visualization of the plot.

The filament length in terms of number of cells, measured for each species at different time points, was first investigated (Figure 1, Table 1 and Figure S1). We show the results of one representative experiment. For each time point, the measurements and statistical analysis comes from two different wells, and three independent microscopy images per well (hence six independent images per time point). Compared to the other investigated species, filaments of *S. salivarius* were distinctively longer. As shown quantitatively in Table 1, the average length of *S. salivarius* reached a peak of 8.9 cells, whereas that of *S. mutans*, *S.* anginosus and *S. oralis* peaked at 4.4, 4.6 and 3.9 cells respectively. Similarly, the maximum length achieved at each time point was markedly higher in *S. salivarius* (50.9 cells) than in the other species (15.1, 16.3 and 18.2 in *S. mutans*, S. anginosus and *S. oralis* respectively). This difference in length of *S. salivarius* has been tested statistically at five time points, corresponding to the starting, early exponential, middle growth, late growth and end phases of each strain (Table 2, exact times indicated in the table). One hour after inoculum, none of the species is significantly different from the other. In all other growth stages tested, *S. salivarius* was significantly different from the other strains, with the only exception of the comparison with *S. anginosus* at the late growth stage. In all species, filament length increased in the exponential population growth phase, and started declining as population size reaches carrying capacity. *S. mutans* and *S. salivarius* show a marked short-long-short pattern, whereby the proportion of short filaments is greatly higher at the inoculum and at carrying capacity than during the exponential phase (Figure 1, panels A and B). Short, medium and long filaments correspond to those made up of 1-2, 3-8 and more than 8 cells in the case of *S. mutans*, *S.* anginosus and *S. oralis*. For *S. salivarius*, the categories correspond to 1-10, 11-30 and more than 30 cells. In the case of *S. anginosus* and *S. oralis*, after the initial increase, the average length drops before reaching the steady value (Figure 1, panels C and D). For all species, long filaments (light grey pie slice, Figure 1) appear or increase mostly during exponential growth phases. This is also confirmed alternatively by the optical density (OD) measurements, plotted below the corresponding average length trend in Figure 1. With the exception of *S. anginosus*, the proportion of small filaments at the inoculum is comparable to that achieved at carrying capacity (black pie slice). A bar plot representing the proportion of short, medium and long filaments of each species at each time point can be found as Figure S1.

An additional aspect investigated in this study was the time kinetics of filament elongation. It was found that filamentation also differed in terms of time scale among the different streptococci (Table 1). *S. mutans* and *S. oralis* reached the maximum average length within 2.2 hours after inoculation. For *S. salivarius* instead, the average filament length peaked at 4 hours, coinciding with a pronounced increase of medium length filaments and the initial appearance of longer ones. In the case of *S. anginosus*, two similar peaks of increased filamentation activity were identified. A first one was in the very early stage of the cultures (1.3 hours, average length of 4.5 cells) and a second one at a later stage (9 hours, average length of 4.6 cells). In the case of *S. mutans*, long filaments become much rarer after 6 hours, and their average length dropped to a value close to that of the steady state (2-3 cells per filament). However an additional 3 hours were required until the mean filament length stabilized at a value of 1.8 cells.

**Figure 2:**
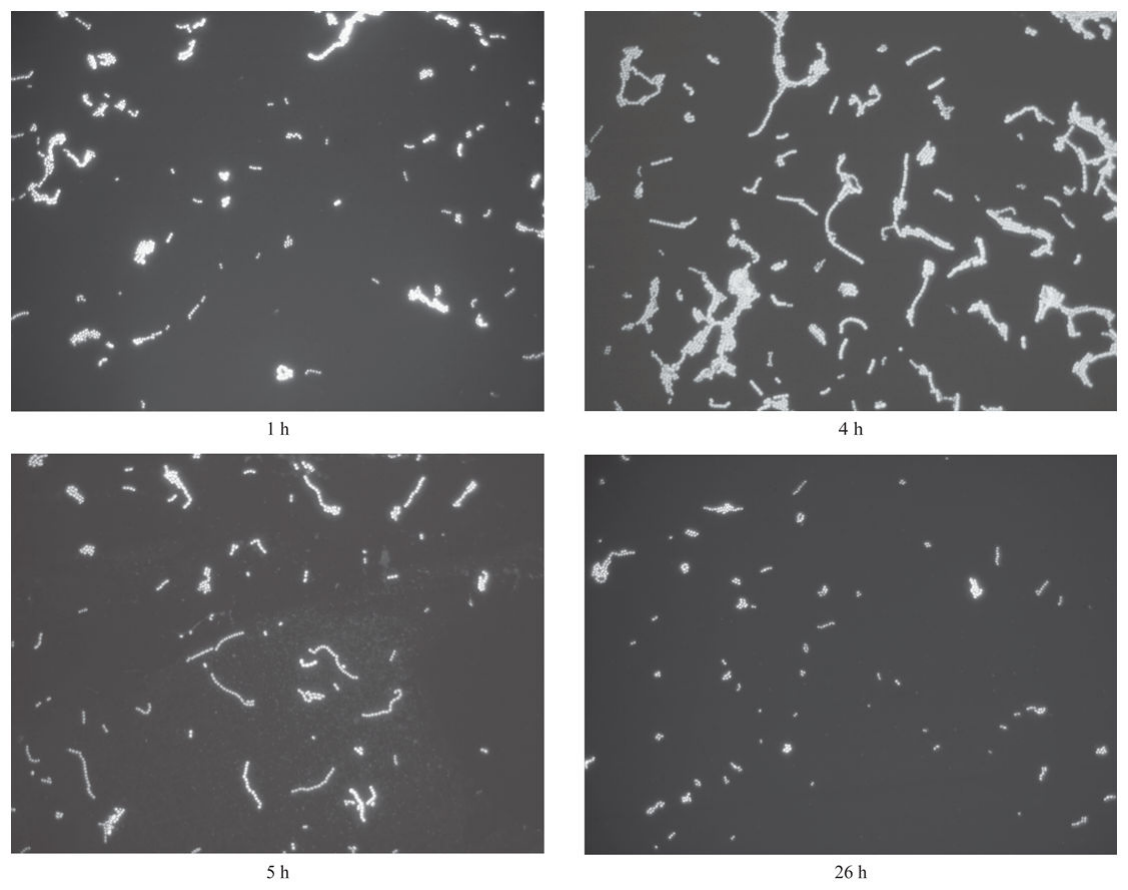
Filaments of *S. salivarius.* The four pictures have been taken from *S*. *salivarius* cultures after 1, 4, 5 and 26 hours after inoculum respectively. While after 4-5 hours filaments lengths are near their maximum, after 26 hours they return to shortened lengths similar to those of the initial phase.

Sample microscopy images of *S. salivarius* at four different growth stages are shown in Figure 2.

## Discussion

The study investigated the filamentation capacity of four well characterized oral streptococci, indicating phenotypic diversity in this respect. This diversity is demonstrated on the established filament length, as well as the kinetics of filamentation activity. These findings are relevant for both evolutionary biology and oral microbiology. In terms of evolution, the findings indicate that filamentation capacities and phenotypes vary not only between genera, but also among species within the same genus, in this case oral streptococci. Hence, phenotypic diversity of filaments is confirmed on species level. These findings prove that the elongation pattern observed in aquatic bacteria [18] can also be present in bacteria inhabiting a completely different habitat, in this case the oral cavity. In particular, *S. mutans* and *S. salivarius* show more markedly the short-long-short pattern of filament length, indicating that regardless of the specific habitat, multicellularity can in principle arise because of the interplay between birth and death rates at the cellular level. Thus, the multicellular life cycle is potentially an emergent property of filamentous bacteria. Still, it should be noted that our findings represent an *in vitro* experimental system, using specific oral species, and under standardized culture conditions.

In the context of oral microbiology, to date there has been no information on the capacities of the different oral streptococci to form filaments. Hence, the present findings may be of importance for the pathophysiology of oral diseases. Streptococci are the predominant genus various aspects of the oral cavity, including tooth surface, oral and tongue mucosa, as well as saliva [22]. The length of filaments may be associated to the capacity of the streptococci to be involved in biofilm formation. For instance, oral streptococci including *S. oralis*, are considered early colonizing bacteria of the tooth surface, and can mediate the adhesion and inclusion of further diverse bacterial species into the biofilm biomass [24]. Moreover, earlier studies have shown an association of streptococcal filaments, for instance of *S. sanguinis*, with the so called “corncob” structures observed by electron microscopy on biofilms isolated from the tooth surface [25], which appear to constitute co-aggregates with fusobacteria [26,27].

In the oral cavity, *S. anginosus* is associated with odontogenic periapical abscesses [28] whereas *S. mutans* is highly associated with dental caries [29]. *S. salivarius* is the predominant commensal species of the oral cavity and highly prevalent on the mucosal surfaces and saliva. It is more associated with oral health than disease, to an extent that contemporary research approaches are considering its usage as a probiotic species [30-32]. What has been markedly different in the case of *S. salivarius* compared to the other oral streptococci studied here is the markedly increased filament length, shown to be statistically significant at four different growth stages (Table 2). This may be required for the better adhesion of this species to the oral mucosa, which is highly movable and continuously shedding, as opposed to the tooth surface. Hence, increased filament length may serve a better “anchoring” advantage of *S. salivarius* within its ecological niche. Since *S. salivarius* is the least disease-associated species compared to the other three streptococci studied here, it is also tempting to postulate that increased filamentation may be a property of commensals, whereas reduced filamentation is a virulence property. Indeed, this interpretation of the present findings is strongly supported by earlier work demonstrating that *S. salivarius* can only weakly invade and kill human endothelial cells compared to a panel of other oral streptococci, including *S. sanguinis*, *S.* oralis and *S. mutans* [33].

## Conclusions

This study indicates that multicellular life cycles are an emergent property of filamentous oral bacteria and that phenotypic differences in filamentation occur within species of the same genus, in this case oral streptococci. Given the potential roles of various oral streptococci in oral health and disease, these results can open new perspectives in the study of the virulence properties of these species. A particularly interesting finding is the higher filament length achieved by *S. salivarius*, compared to the other oral streptococci studied. As this species is considered the least disease-associated, there is merit to investigate further if a long filamentation is associated with decreased bacterial virulence.

## Supporting Information

Figure S1 **Bar plot representing the proportion of short, medium and long filaments of each species at each time point.**
(TIF)
