# Supplementary figures and images for: Phenotypic Diversity of Multicellular Filamentation in Oral Streptococci
Source: PLoS One. 2013 Sep 27;8(9):e76221. doi: 10.1371/journal.pone.0076221 (PMC3785443; doi:10.1371/journal.pone.0076221)

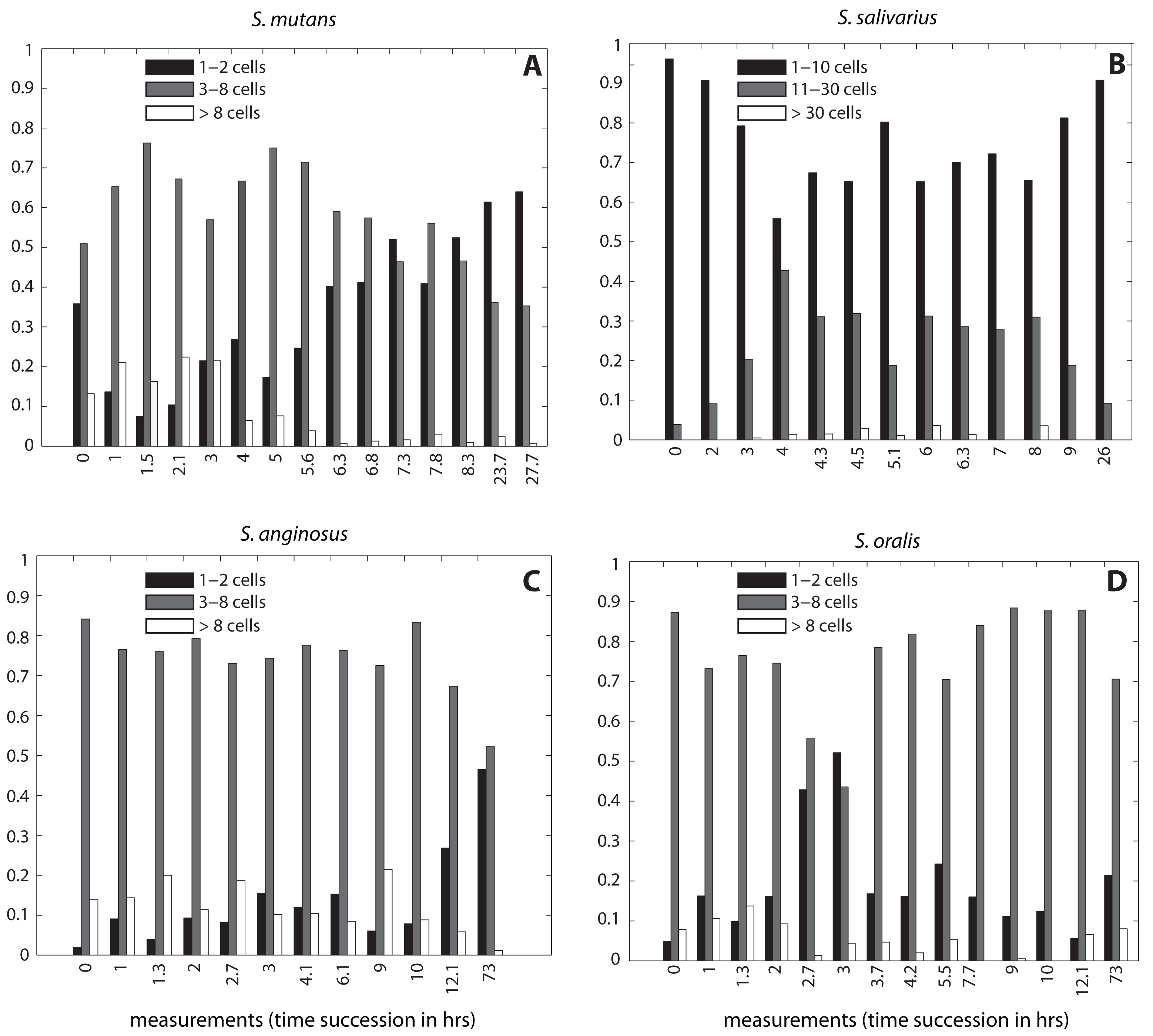

Supplement: Figure S1 — Bar plot representing the proportion of short, medium and long filaments of each species at each time point. (TIF) [file pone.0076221.s001.tif]
